# Supplementary material for: Deletion of wfs1 Impairs Oligodendrocyte Precursor Cells Dorsal Distribution and Myelination Through the wfs1-hmgcs1 Axis in Zebrafish
Source: Biology (Basel). 2026 Aug 16;15(16):1402. doi: 10.3390/biology15161402 (PMC13510151; doi:10.3390/biology15161402)
Supplement: Supplementary file 1 [file biology-15-01402-s001.zip › File S1-Transcriptome sequencing data and differentially expressed genes (DEGs) in wfs1-- zebrafish at 5 dpf/2.KEGG/wfs1KOvsWT/ALL/src/dre01040.html]

dre01040


- dre:140615

- Up regulated genes

140615(1.00910734824653)

- dre:140615

- Up regulated genes

140615(1.00910734824653)

- dre:140615

- Up regulated genes

140615(1.00910734824653)

- dre:393441

- Up regulated genes

393441(1.51625055086835)

- dre:317738

- Down regulated genes

317738\_1(-2.49636387798148)

- dre:393441

- Up regulated genes

393441(1.51625055086835)

- dre:334217

- Down regulated genes

334217(-1.85360471365525)

- dre:393441

- Up regulated genes

393441(1.51625055086835)

- dre:334217

- Down regulated genes

334217(-1.85360471365525)

- dre:393441

- Up regulated genes

393441(1.51625055086835)

- dre:334217

- Down regulated genes

334217(-1.85360471365525)

- dre:393441

- Up regulated genes

393441(1.51625055086835)

- dre:140615

- Up regulated genes

140615(1.00910734824653)

- dre:140615

- Up regulated genes

140615(1.00910734824653)

- dre:393441

- Up regulated genes

393441(1.51625055086835)

- dre:393441

- Up regulated genes

393441(1.51625055086835)

- dre:393441

- Up regulated genes

393441(1.51625055086835)

- dre:317738

- Down regulated genes

317738\_1(-2.49636387798148)

- dre:393441

- Up regulated genes

393441(1.51625055086835)

- dre:393441

- Up regulated genes

393441(1.51625055086835)

- dre:393441

- Up regulated genes

393441(1.51625055086835)

- dre:140615

- Up regulated genes

140615(1.00910734824653)

- dre:140615

- Up regulated genes

140615(1.00910734824653)

- dre:393441

- Up regulated genes

393441(1.51625055086835)

- dre:554145

- Up regulated genes

554145(1.02117229708517)

- dre:393441

- Up regulated genes

393441(1.51625055086835)

- dre:554145

- Up regulated genes

554145(1.02117229708517)

- dre:393441

- Up regulated genes

393441(1.51625055086835)

- dre:554145

- Up regulated genes

554145(1.02117229708517)

- dre:393441

- Up regulated genes

393441(1.51625055086835)

- dre:554145

- Up regulated genes

554145(1.02117229708517)

- dre:393441

- Up regulated genes

393441(1.51625055086835)

- dre:554145

- Up regulated genes

554145(1.02117229708517)

- dre:393441

- Up regulated genes

393441(1.51625055086835)

- dre:554145

- Up regulated genes

554145(1.02117229708517)

- dre:393441

- Up regulated genes

393441(1.51625055086835)

- dre:554145

- Up regulated genes

554145(1.02117229708517)

- dre:393441

- Up regulated genes

393441(1.51625055086835)

- dre:140615

- Up regulated genes

140615(1.00910734824653)

- dre:317738

- Down regulated genes

317738\_1(-2.49636387798148)

- dre:393441

- Up regulated genes

393441(1.51625055086835)

- dre:140615

- Up regulated genes

140615(1.00910734824653)

- dre:317738

- Down regulated genes

317738\_1(-2.49636387798148)

- dre:393441

- Up regulated genes

393441(1.51625055086835)

- dre:393441

- Up regulated genes

393441(1.51625055086835)

- dre:334217

- Down regulated genes

334217(-1.85360471365525)

- dre:393441

- Up regulated genes

393441(1.51625055086835)

- dre:334217

- Down regulated genes

334217(-1.85360471365525)

- dre:393441

- Up regulated genes

393441(1.51625055086835)

- dre:393441

- Up regulated genes

393441(1.51625055086835)

- dre:554145

- Up regulated genes

554145(1.02117229708517)

- dre:393441

- Up regulated genes

393441(1.51625055086835)

- dre:554145

- Up regulated genes

554145(1.02117229708517)

- dre:393441

- Up regulated genes

393441(1.51625055086835)

- dre:393441

- Up regulated genes

393441(1.51625055086835)

- dre:393441

- Up regulated genes

393441(1.51625055086835)

- dre:554145

- Up regulated genes

554145(1.02117229708517)

- dre:393441

- Up regulated genes

393441(1.51625055086835)

Close
